# Supplementary material for: sEMG-Based Hand Posture Recognition and Visual Feedback Training for the Forearm Amputee
Source: Sensors (Basel). 2022 Oct 19;22(20):7984. doi: 10.3390/s22207984 (PMC9608765; doi:10.3390/s22207984)
Supplement: Supplementary file 1 [file sensors-22-07984-s001.zip › sensors-1943080-supplementary.pdf]

Supplementary Materials

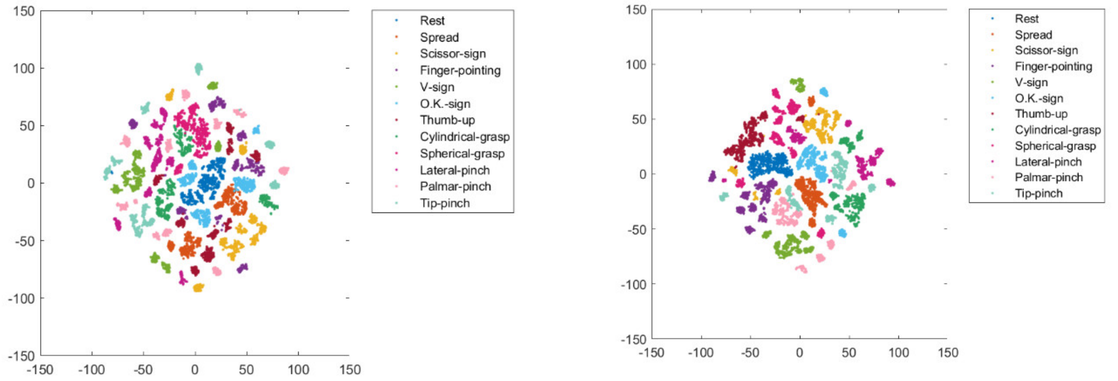

(a) (b)

Figure S1. t-SNE visualization of variability in the sEMG signals of a healthy adult (subject 2) (a) Day 1, (b) Day 2.

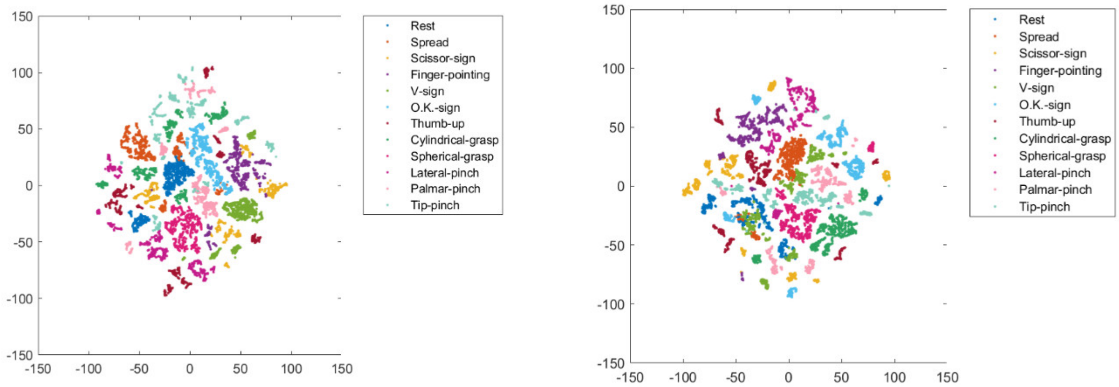

(a) (b)

Figure S2. t-SNE visualization of variability in the sEMG signals of a healthy adult (subject 3) (a) Day 1, (b) Day 2.

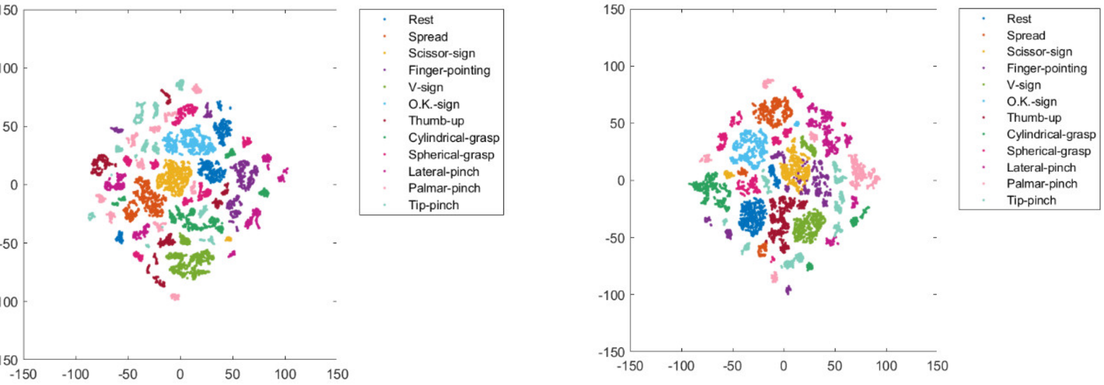

(a) (b)

Figure S3. t-SNE visualization of variability in the sEMG signals of a healthy adult (subject 4) (a) Day 1, (b) Day 2.

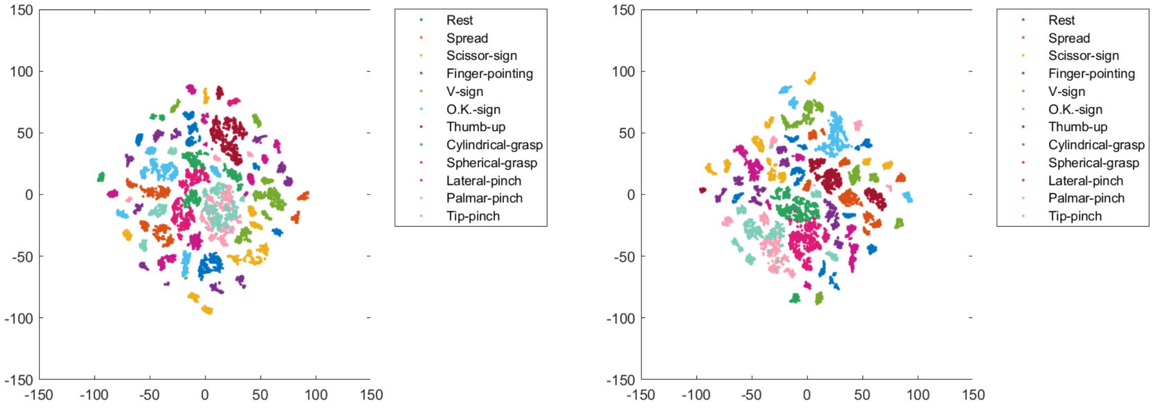

(a) (b)

Figure S4. t-SNE visualization of variability in the sEMG signals of a healthy adult (subject 5) (a) Day 1, (b) Day 2.

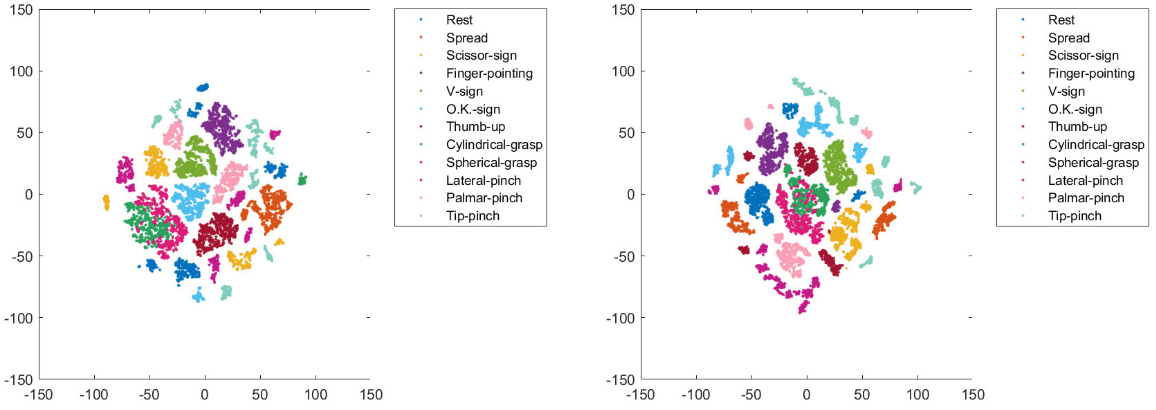

(a) (b)

Figure S5. t-SNE visualization of variability in the sEMG signals of a healthy adult (subject 6) (a) Day 1, (b) Day 2.

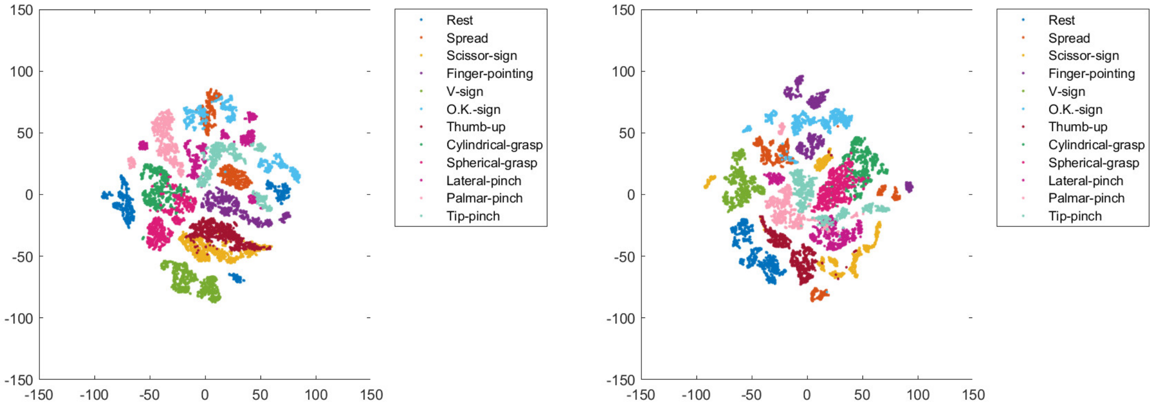

(a) (b)

Figure S6. t-SNE visualization of variability in the sEMG signals of a healthy adult (subject 7) (a) Day 1, (b) Day 2.

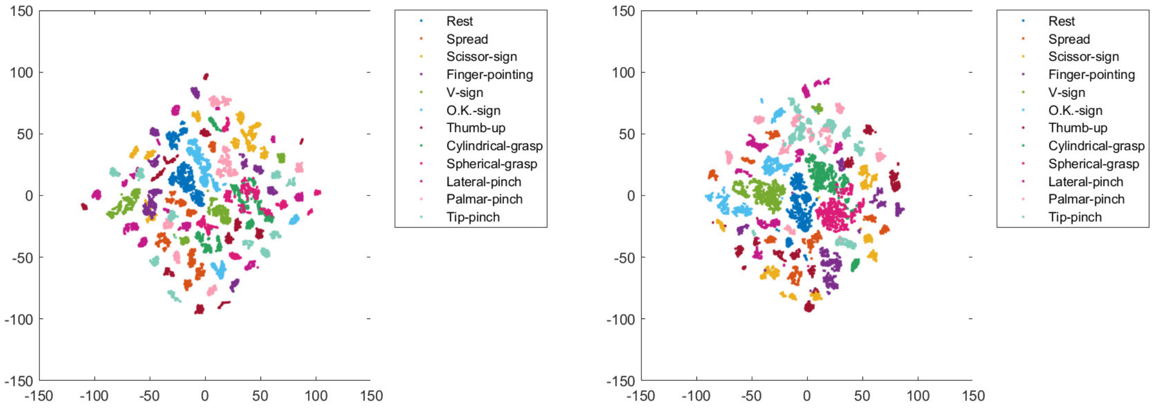

(a) (b)

Figure S7. t-SNE visualization of variability in the sEMG signals of a healthy adult (subject 8) (a) Day 1, (b) Day 2.

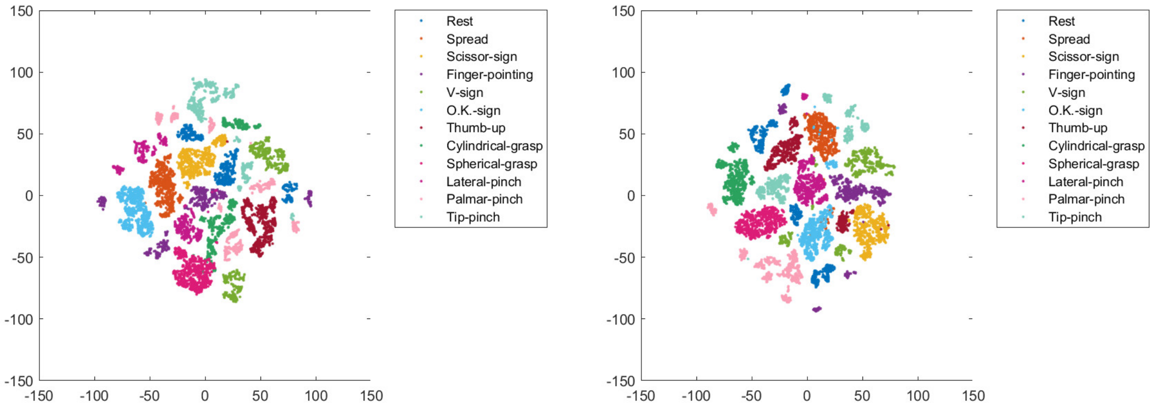

(a) (b)

Figure S8. t-SNE visualization of variability in the sEMG signals of a healthy adult (subject 9) (a) Day 1, (b) Day 2.

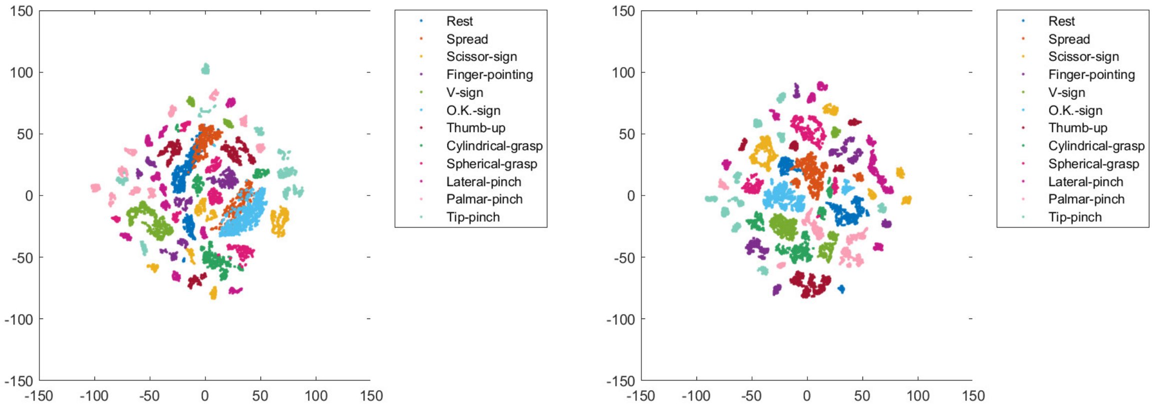

(a) (b)

Figure S9. t-SNE visualization of variability in the sEMG signals of a healthy adult (subject 10) (a) Day 1, (b) Day2.
